# CREB3 Transcription Factors: ER-Golgi Stress Transducers as Hubs for Cellular Homeostasis

**DOI:** 10.3389/fcell.2019.00123

**Published:** 2019-07-03

**Authors:** Luciana Sampieri, Pablo Di Giusto, Cecilia Alvarez

**Affiliations:** ^1^Centro de Investigaciones en Bioquímica Clínica e Inmunología (CIBICI-CONICET), Córdoba, Argentina; ^2^Departamento de Bioquímica Clínica, Facultad de Ciencias Químicas, Universidad Nacional de Córdoba, Córdoba, Argentina

**Keywords:** CREB3 transcription factors, ER stress response, Golgi stress response, secretory pathway, secretory capacity, cellular homeostasis, central nervous system

## Abstract

CREB3 family of transcription factors are ER localized proteins that belong to the bZIP family. They are transported from the ER to the Golgi, cleaved by S1P and S2P proteases and the released N-terminal domains act as transcription factors. CREB3 family members regulate the expression of a large variety of genes and according to their tissue-specific expression profiles they play, among others, roles in acute phase response, lipid metabolism, development, survival, differentiation, organelle autoregulation, and protein secretion. They have been implicated in the ER and Golgi stress responses as regulators of the cell secretory capacity and cell specific cargos. In this review we provide an overview of the diverse functions of each member of the family (CREB3, CREB3L1, CREB3L2, CREB3L3, CREB3L4) with special focus on their role in the central nervous system.

## Introduction

Eukaryotic cells have different ways of achieving homeostasis and coping with cellular requirements. The secretory pathway plays a fundamental role in maintaining homeostasis since it needs to adapt to endogenous and exogenous stimuli to regulate the cellular capacity for secretion. Most of the studies aimed to understanding the adaptation of the secretory pathway have been carried out inducing some type of organelle-specific stress, such as cargo overload, structural damage or perturbation of an enzymatic activity. Consequently, the signaling pathways activated to achieve homeostasis are considered a response to stress. In this sense, the ER stress response (or the unfolded protein response) and the Golgi stress response have been described. In addition, lysosomes and peroxisomes have their own stress responses, and a mitochondrial unfolded protein response (UPRmt) has also been reported (Sasaki and Yoshida, 2015; Melber and Haynes, 2018). In general, a stress response is triggered by a sensor protein, that detects the insufficiency of organelle function and activates one or more transcription factors which in turn induce the transcription of genes involved in the modulation of organelle function. For instance, to upregulate the capacity of the ER, sensor molecules located on the ER membrane, such as ATF6, IRE1 and PERK, activate transcription factors which increase the transcription of ER-related genes (Walter and Ron, 2011).

The Golgi stress response is triggered when its capacity to handle protein processing is overloaded and the cell needs to increase the expression of modification enzymes. This response, which has been less studied than the ER stress response, is associated with the following pathways: TFE3 (Taniguchi et al., 2015), HSP47 (Miyata et al., 2013), proteoglycans (PG) (Sasaki et al., 2019), ETS (E26 transformation-specific, Baumann et al., 2018), and CREB3 (Reiling et al., 2013). Oku et al. (2011) and Taniguchi et al. (2015, 2016) have identified that TFE3 and MLX transcription factors regulate expression of some Golgi-related genes after inhibiting Golgi function with monensin, among other treatments. TFE3 and MLX bind to the *cis*-acting element called the Golgi apparatus stress response element (GASE) and modulate the TFE3 pathway. MLX competes with TFE3 for GASE sites resulting in the attenuation of TFE3 induction. The HSP47 pathway was characterized using a GALNAc structural analog (named BG or GalNAc-bn) which is a competitive inhibitor of mucin type *O*-glycosylation. This treatment induces expression of the ER chaperone HSP47 that prevents Golgi stress-induced apoptosis (Miyata et al., 2013). It is not clear how this ER-localized chaperone evades BG-induced Golgi stress. Moreover, cellular treatments performed to reduce PG glycosylation in the Golgi (Sasaki et al., 2019) contributed to characterize the PG pathway, which regulates the expression of PG-induced Golgi stress genes. Furthermore, Baumann et al. (2018) have recently identified three ETS transcription factor family members, ELK1, ETS1 and GABPA/B that respond to pharmacological Golgi disruption, suggesting that they operate in parallel. In addition to the TFE3, HSP47, PG, and ETS pathways, Reiling et al. (2013) reported the CREB3 pathway that leads to Golgi stress response, inducing apoptosis through ARF4 transcriptional activation after Brefeldin A treatment. CREB3 belongs to the CREB3 family of transcription factors that were shown to regulate numerous genes involved in secretory capacity and structure of the Golgi complex, including ER chaperones and various transport factors (Bailey and O’hare, 2007; Murakami et al., 2009; Fox et al., 2010; Barbosa et al., 2013). The study of transcription factors that respond to Golgi stress as well as the resulting signaling pathways are essential to understand the role of this organelle in physiological and pathological contexts such as tumor and neurodegenerative processes. In this review we provide an overview of the CREB3 family, and details of the individual roles of all members (CREB3, CREB3L1, CREB3L2, CREB3L3, CREB3L4) with special focus on CREB3, CREB3L1, and CREB3L2 functions in the central nervous system (CNS).

## Overview of the CREB3 Family

The CREB3 family of transcription factors is comprised of five members in mammals: CREB3, CREB3L1, CREB3L2, CREB3L3, and CREB3L4. They belong to the large bZIP family, which is one of the mayor families of transcription factors. CREB3 family is highly related to the S terol R egulatory E lement-B inding P roteins (SREBPs) and the A ctivating T ranscription F actor 6, ATF6, families. They also have leucine zipper domain and undergo regulated intramembrane proteolysis (RIP). SREBPs and ATF6 are prototypical ER-bound transcription factors that act in response to different signals recognized by the ER. SREBPs regulate fatty acid and cholesterol metabolism. The stimulus for SREBPs activation is the absence of sterols, and its N-terminal domain promotes transcription of many genes involved in cholesterogenesis and lipogenesis (Eberle et al., 2004). ATF6 is best known for its role in transducing signals linked to ER stress (Haze et al., 1999). Recent reports, however, have described novel functions for ATF6 related to organogenesis and tissue homeostasis (Wang et al., 2015; Naranjo et al., 2016; Jin et al., 2017). ATF6 is retained in the ER through interactions between its luminal tail and the ER chaperone GRP78/BiP. ER stress induces GRP78/BiP dissociation from ATF6, resulting in the exposure of its Golgi-localization sequences (Shen et al., 2002). Following translocation to the Golgi Complex, ATF6 is cleaved by site-1 protease (S1P) and site-2 protease (S2P) to release its N-terminal active fragment. This active portion of ATF6 is then transported to the nucleus, where it binds to ER stress-response elements which results in the expression of ER stress proteins including GRP78/BiP and XBP1 (Hillary and Fitzgerald, 2018).

The overall range of functions of the CREB3 family includes development, metabolism, secretion, survival, differentiation, tumorigenesis and cell division, among others. One important finding regarding the role of CREB3 transcription factors in secretion was reported by Fox et al. (2010). They identified in the Drosophila salivary gland factors required for its secretory function and found that dCREB-A (the only CREB3 family member encoded by Drosophila) is required to enhance the expression of genes encoding components of the secretory pathway. Moreover, they showed that dCREB-A also targets genes encoding cell specific proteins that require the secretory pathway to reach their destination.

**FIGURE 1:**
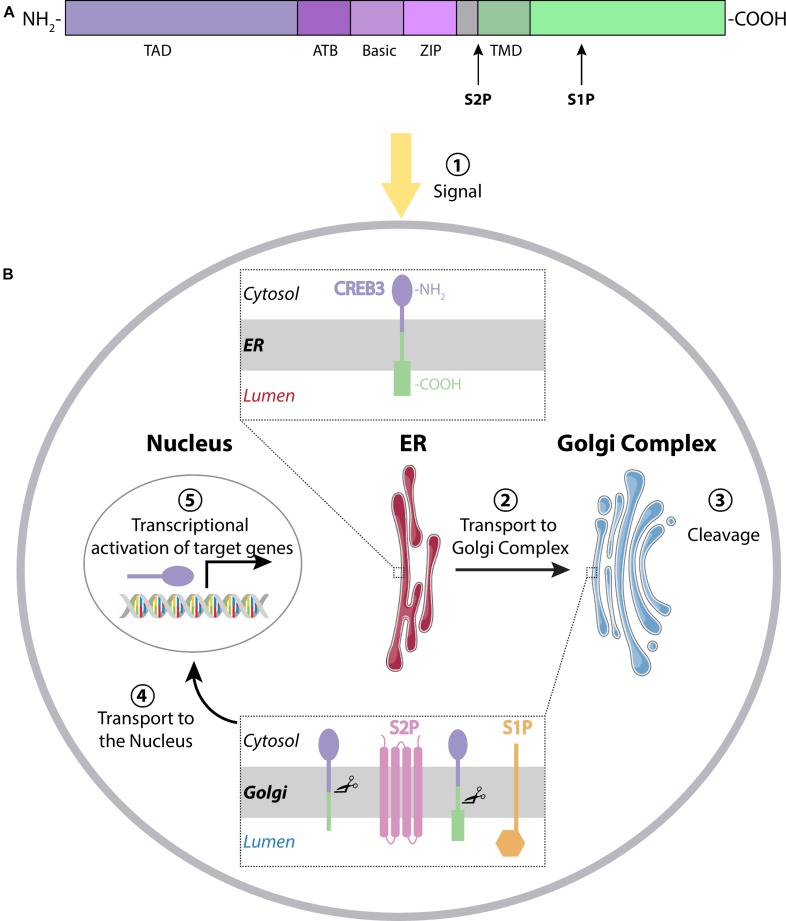
**(A)** CREB3 family members have a characteristic domain next to the Transactivation Domain (TAD), the adjacent to bZip Domain (ATB). The basic region, the leucine zipper and the transmembrane domain (TMD) are shown in different colors. Cleavage sites for S1P and S2P are indicated with arrows. **(B)** Mechanism of action: Transcription factors of the CREB3 family reside in the endoplasmic reticulum (ER) in their inactive form. After stimulation (1), they are transported from the ER to the Golgi Complex (2), where they are cleaved by S1P and S2P proteases (3) to release the N-terminal fragment. This active form then translocates to the nucleus (4) to activate transcription of their target genes (5).

CREB3 transcription factors are single-pass membrane proteins localized in the ER with their N-terminus facing the cytoplasm and the C-terminus the ER lumen (Figure 1). Details of the sequence homologies between the different members of the group have been previously reviewed (Chan et al., 2011; Fox and Andrew, 2015). In summary, CREB3 members share the following functional domains (named from N- to C-terminus, Figure 1A): the transactivation domain (TAD) that mediates sequence specific DNA binding, a conserved domain of approximately 30 residues called ATB (adjacent to bZIP), a basic region (Basic) next to the leucine zipper domain (Zip) called together bZIP DNA-binding domain, and a transmembrane domain (TMD). The ATB domain is not part of the bZIP, but a distinct feature of this family and may consequently indicate special functions for these proteins (Figure 1A, Bailey and O’hare, 2007). In response to different signals, including ER stress, CREB3 proteins are transported from the ER to the Golgi complex where they are cleaved (activated) through RIP (Brown et al., 2000) by S1P and S2P proteases sequentially. The first cleavage is performed by S1P, a membrane-bound serine protease of the subtilisin family (Sakai et al., 1998). After that, the resulting CREB3 protein is cleaved by S2P, a membrane-embedded zinc metalloprotease to release the N-terminal fragment, which translocates into the nucleus and activates the transcription of target genes (Figure 1B). Also, the N-terminal form of CREB3 transcription factors can form homo- and hetero-dimers with differential transcriptional activity (Vinson et al., 2006).

CREB3 transcription factors can be regulated by their proteolytic cleavage (induced by both ER and Golgi signals) and by increase of their transcription. Transcription of dCREB-A is cooperatively regulated by the transcription factors CBP (CREB-binding protein) and Cut (Chung et al., 2017). Interestingly, in dendritic cells, expression of constitutively active form of CREB3 increases its own transcript levels (Sanecka et al., 2012). In chondrocytes, CREB3L2 is a direct target gene of the master regulator for chondrogenesis, Sox9 (Hino et al., 2014) and orphan nuclear receptor Nr4a1 controls the expression of CREB3L1 in Arginine vasopressin neurons (Greenwood et al., 2017). Ceramide also acts as a CREB3L1 upstream regulator by promoting its proteolytical activation (Denard et al., 2012). Despite the identification of some signals and molecules that lead to the activation of members the CREB3 family, their regulation in different tissues remains poorly studied. The dual localization of CREB3 transcription factors (ER and Golgi complex) and their ability to sense ER stress and to respond to Golgi disruption posit them as molecules involved in both the ER and Golgi stress response.

## Creb3

CREB3, also called LZIP or Luman (after a legendary hero in ancient China; Lu et al., 1997), was identified through yeast-two hybrid assays for its interaction with the transcriptional co-activator HCF (Host Cell Factor, Freiman and Herr, 1997; Lu et al., 1997). CREB3 mRNA is detected in many rat and human tissues, but liver and nervous system have the highest levels of expression (Ying et al., 2015).

CREB3 levels increase in an age-specific manner in Leydig cells, which are responsible for the synthesis of testosterone in testicles. CREB3 knock-down in mouse testis leads to an increase in steroidogenesis genes expression and testosterone synthesis (Wang et al., 2019). In contrast, in ovarian mouse granulosa cells, CREB3 depletion induces a decrease of estradiol and progesterone synthesis and promotes cell proliferation (Zhao et al., 2016). Steroidogenic enzymes and cell cycling factors-encoding genes were found down-regulated and up-regulated in CREB3 knock-down cells, respectively. These results highlight an important role of CREB3 in male and female reproduction.

In agreement with data found in Drosophila CREB3 gene, dCREB-A, expression of the constitutive active CREB3 form in dendritic cells induces up-regulation of secretory pathway genes, including COPII components as well as Golgi proteins such as GBF1 and Arf4 (Sanecka et al., 2012). In line with these results, a CREB3 isoform lacking the transmembrane domain induces ARF4 transcription by binding to the cAMP response element motif in the ARF4 promoter (Jang et al., 2012). Moreover, the CREB3-ARF4 signaling is implicated in the Golgi stress response induced by BFA treatment and supports the survival of *Chlamydia trachomatis* and *Shigella flexneri* (Reiling et al., 2013). Moreover, the *Chlamydia pneumoniae*-specific inclusion membrane protein, Cpn0147, interacts with CREB3 to mediate the interaction with the host cell endoplasmic reticulum (Zhao et al., 2017).

It has been reported that in human osteogenic sarcoma cells (HOS) CREB3 binds to the CC chemokine receptor 1 (CCR1) and participates in Leukotactin-1-induced cell migration by enhancing the NF-κB activation pathway (Ko et al., 2004; Jang et al., 2007a, b). Furthermore, Sung et al. (2008) showed that CREB3 regulates the expression of the chemokine receptors CCR1 and CCR2 in a human monocyte cell line (THP-1). Interestingly, these two receptors are involved in the early stages of atherogenesis. Altogether, these data account for an important function of CREB3 in the process of cell migration with high impact on the pathogenesis of atherosclerosis. Additionally, CREB3 expression has been linked to breast cancer. In both MCF-7 and MD-MB-231 breast cancer cell lines, CREB3 binds specifically to Histone Deacetylase 3 (HDAC3). In this context, HDAC3 is a co-repressor of CREB3-mediated CXCR4 gene expression (Kim et al., 2010). *CXCR4* is a chemokine receptor, a target of CREB3 and a crucial mediator of cell migration in both leukocytes and tumor cells. Interestingly, CXCR4 is highly expressed in primary and metastatic human breast cancer cells. Howley et al. (2018) found that ARF4, COPB1 and USO1, ER-Golgi trafficking proteins regulated by CREB3, are associated with an invasive phenotype in a mouse model of metastatic progression. This evidence points out to the relevance of the regulation of components of the secretory pathway, such as the Golgi Complex, by members of the CREB3 family not only in physiological contexts but also in a pathologic environment such as cancer.

## CREB3L1

CREB3L1 was originally named OASIS (for old astrocyte specifically induced substance) because it was identified in a screening for genes induced in long-term cultured astrocytes (old astrocytes) obtained from newborn mice brains as an *in vitro* model to study gliosis (Honma et al., 1999). Northern blot assays performed in multiple human tissues indicated that heart, placenta, pancreas, prostate, lung, and colon express higher CREB3L1 levels than brain, testis, and skeletal muscle (Omori et al., 2002). CREB3L1 is also highly expressed in osteoblasts. In fact, CREB3L1-deficient mice exhibited severe osteopenia caused by a decrease in the levels of type I collagen, the major component of the bone matrix (Murakami et al., 2009). In agreement with that, CREB3L1 activates transcription of type I collagen a1 gene, *Col1a1*, by directly binding to a CRE-like sequence in its promoter region. Other targets of CREB3L1 are *Xbp1* and the chaperone protein GRP78/BiP, genes typically up-regulated during ER stress (Murakami et al., 2009). Moreover, CREB3L1 regulates bone angiogenesis during bone development regulating the expression of hypoxia-inducible factor-1α (HIF-1α) target genes (Cui et al., 2015). CREB3L1 critical contribution in bone formation was also confirmed by its role as a genetic cause of autosomal recessive osteogenesis imperfecta in humans (Symoens et al., 2013; Keller et al., 2018; Guillemyn et al., 2019). CREB3L1 mutant forms identified in these patients also down-regulate the expression of COPII components, Sec23A and Sec24D.

CREB3L1 expression was detected in pancreatic beta-cell lines and rodent islets and is highly active during pancreas development. Transfection of active form of CREB3L1 in a pancreatic β-cell line induced expression of genes involved in protein transport and implicated extracellular matrix production (Vellanki et al., 2010). Results from our group indicate that, in thyroid cells, CREB3L1 levels are up-regulated by thyrotropin and CREB3L1 is sufficient to increase transport proteins levels and induce Golgi enlargement (Garcia et al., 2017). We also show that expression of CREB3L1 dominant negative hampers the TSH-induced Golgi enlargement. Moreover, CREB3L1 expression is frequently altered in many cancer types and in some of them, like in breast and bladder cancer, is epigenetically silenced through DNA methylation (Rose et al., 2014; Ward et al., 2016). Interestingly, a retrospective study performed on biopsy samples analysis from triple negative breast cancer indicated that CREB3L1 levels in tumors responsive to doxorubicin chemotherapy were significantly higher than in those resistant to this treatment (Denard et al., 2018). It has been postulated that CREB3L1 is a metastasis suppressor and that it may function in a similar way to p53 as a regulator of cell proliferation (Denard et al., 2011). In contrast, CREB3L1 is up-regulated in a metastatic subtype of triple-negative breast cancer cells that have activated both PERK signaling and the epithelial-to-mesenchymal transition program (Feng et al., 2017). In this tumor subtype CREB3L1 expression promotes invasion through the activation of extracellular matrix genes such as Col1a and FN1.

## CREB3L2

CREB3L2 also known as BBF2 human homolog on chromosome 7 (BBF2H7) was identified as a novel human protein whose C-terminal region is fused to the FUS (fusion) genes in low-grade fibromyxoid sarcoma as a result of chromosomal translocation. Changes in CREB3L2 levels were first described in C6 glioma, HEK293 and MEF cells treated with thapsigargin (Kondo et al., 2007). Moreover, CREB3L2 mRNA levels were detected in different cell types and tissues (Kondo et al., 2007; Panagopoulos et al., 2007).

One of the findings that link CREB3L2 to the regulation of the secretory pathway is its participation in the differentiation of hepatic stellate cells (HSCs) to myofibroblast-like cells, a critical event in hepatic fibrosis. This process is characterized by enlargement of the ER and Golgi complex. Interestingly, another feature of this process is the up-regulation of Sec23A and Sec24D, components of the coat protein complex II (COPII), mediated by CREB3L2 (Tomoishi et al., 2017).

CREB3L2 mRNA is enriched in the developing notochord of *Xenopus laevis* embryos, where it also regulates genes of the secretory pathway (Tanegashima et al., 2009). Moreover, during early embryonic development of medaka fish, CREB3L2 is required for transcriptional regulation of a complete set of genes (*Sec23a*/*24d*/*13*/*31a*, *Tango1*, *Sedlin*, and *KLHL12*) essential for the enlargement of COPII vesicles to accommodate type II collagen for export from the ER (Ishikawa et al., 2017). In agreement with this, the well described zebrafish *feelgood* mutation, which disrupts head skeleton and notochord development through loss of secretory capacity, consists of a missense mutation in the DNA-binding domain of CREB3L2 and this results in decreased expression of *sec23a* and *sec24d* genes (Melville et al., 2011). An interesting fact about CREB3L2 is that, in developing cartilage, both the N- and C-terminal of the protein have important roles. After CREB3L2 cleavage, the N- terminus exerts its activity as transcription factor by promoting secretion of extracellular matrix proteins through induction of Sec23a expression (Saito et al., 2009; Hino et al., 2014). The C- terminal part of the protein, on the other hand, promotes the proliferation of chondrocytes and inhibits hypertrophic differentiation via regulating the Indian hedgehog (Ihh)/parathyroid hormone-related protein signaling pathway (Saito et al., 2014). CREB3L2, as well as some cartilage matrix genes like *Col2a1*, are targets of Sox9 (Hino et al., 2014). The result of this transcriptional activation axis is the acceleration of cartilage matrix protein secretion during chondrocyte differentiation. In chondrocytes, CREB3L2 also functions as a target of the F-Box protein Fbxw7, a component of SKP1-CUL1-F-box protein type ubiquitin ligase which contributes to stem cell maintenance and cell differentiation. Fbxw7 targets the nuclear form of CREB3L2 for degradation in mesenchymal cells, thereby contributing to chondrogenesis (Yumimoto et al., 2013). Data obtained by Al-Maskari et al. (2018) showed that CREB3L2 expression increases during human B-cell transition to antibody secreting cells, which is a logic finding considering that this event implies great secretory overload (Shi et al., 2015) and that CREB3L2 has already been reported to up-regulate genes involved in the secretory pathway in similar cellular contexts.

The role of CREB3L2 in cancer is mainly represented by the specific translocation t(7;16)(q33;p11) that results in the creation of the chimeric gene FUS-CREB3L2, which is responsible for low-grade fibromyxoid sarcoma (LGFMS); a rare, slow-growing type of cancer that usually forms in the deep soft tissues of the legs or trunk (chest and abdomen) (Panagopoulos et al., 2007; Bartuma et al., 2010). In this context, the chimeric gene is believed to regulate CD24 (Moller et al., 2011). It was also described that, in malignant glioma, an FRS2/PAK1-activated RAS/MAPK signaling cascade up-regulates CREB3L2, which directly binds to the ATF5 promoter resulting in ATF5 transcription, an anti-apoptotic factor which plays a role in cell survival (Sheng et al., 2010).

## CREB3L3

CREB3L3 (also known as CREB-H) was originally isolated as a transcription factor expressed in a liver-specific manner (Omori et al., 2001). CREB3L3 is also expressed in the stomach and small intestine. The roles for this transcription factor include triglyceride metabolism in the liver (Lee et al., 2011), reduction of cholesterol absorption (Kikuchi et al., 2016), glucose and lipid metabolism (Nakagawa et al., 2016b; Nakagawa and Shimano, 2018), acute phase response activation (Zhang et al., 2006) and hepcidin-mediated iron metabolism (Vecchi et al., 2009). CREB3L3 expression is regulated by a number of nuclear receptors, including PPARα (Danno et al., 2010), HNF4α (Luebke-Wheeler et al., 2008), GR (Lee et al., 2010), and ERRγ (Misra et al., 2014).

Surprisingly, CREB3L3 knock-out mice are viable, fertile, have a normal lifespan and do not display any gross physical or behavioral abnormalities. No anatomical or histological differences were found between liver and gastrointestinal tract of CREB3L3−/− mouse embryos respect to the controls. This information argues in favor of the notion that CREB3L3 is not essential for hepatogenesis and hepatocyte differentiation in the mouse. However, CREB3L3−/− mice showed a strong decrease in the transcript levels of acute phase genes compared to control animals when tunicamycin was administered to induce ER stress (Luebke-Wheeler et al., 2008). When intestinal CREB3L3 knock-out mice were compared to floxed mice, there were no apparent differences in metabolic parameters. On the other hand, the liver CREB3L3 knock-out mice showed hyperlipidemia due to increased expression levels of genes related to cholesterol synthesis relative to floxed mice (Nakagawa et al., 2016a). Moreover, a whole genome expression analysis performed recently on liver samples from CREB3L3−/− mice subjected to ketogenic diet underscored the relevance of CREB3L3 in regulating apolipoprotein metabolism (Ruppert et al., 2019).

It has been demonstrated that CREB3L3 is required for the acute inflammatory response by regulating transcription of CRP and SAP genes which encode C-reactive protein and serum amyloid P-component proteins, respectively. Moreover, CREB3L3 and ATF6 interact with each other to synergistically activate expression of their target genes upon ER stress (Zhang et al., 2006). CREB3L3 has also been strongly linked to the regulation of cell proliferation. Interestingly, it is significantly underexpressed in hepatocellular carcinoma tissues and cells. Also, the loss of CREB3L3 function in hepatocellular carcinoma might contribute to the initiation and/or progression of cancer (Chin et al., 2005).

## CREB3L4

CREB3L4, also known as AIbZIP, CREB4 or TISP40, was first described in 2002 by two independent research groups. One of these groups was originally interested in identifying androgen-regulated genes in human prostate cancer cells, and so CREB3L4 cDNA was isolated from LNCaP human prostate cancer cells treated with the synthetic androgen R1881. Because the cDNA analysis revealed that it contains a region with extensive similarity to the bZIP domain of CREB/ATF transcription factors, the protein was designated AIbZIP (Androgen-Induced bZIP protein; Qi et al., 2002).

The main function of CREB3L4 appears to be related to the tissue where it is mostly expressed; the prostate. This transcription factor has been shown to be involved in the proliferation of prostate cancer cells promoted by the Androgen Receptor (AR) and IRE1α (Kim et al., 2017). Interestingly, it was described that CREB3L4 can interact with CREB3L1 to inhibit its nuclear translocation in LNCaP cells. This results in p21 suppression and, consequently, increase in cell proliferation (Cui et al., 2016). Also, CREB3L4 has been linked to the process of cellular differentiation into adipocytes (Kim et al., 2014). Another group has reported its participation in male germ cell development, underlying that whereas CREB3L4 knock-down moderately impairs spermatogenesis, it is not sufficient to produce infertility in mice (Adham et al., 2005). Downstream target genes of CREB3L4 are tightly associated with prostate cell proliferation. In this context, the direct interaction between CREB3L4 and AR has been reported. Ben Aicha et al. (2007) demonstrated that a number of different genes with diverse functions are induced by CREB3L4: transcription factors, genes involved in protein processing, genes encoding channels and transporters, genes in charge of lipid and sugar metabolism and signal transduction genes, among others. These data imply once again the concept that the functions of CREB3 proteins, in this case CREB3L4, might not only be limited to the response to ER stress.

CREB3L4 is predominantly expressed in prostatic tissue and in breast cancer and prostate cancer cell lines. Interestingly, its expression is higher in cancerous prostate cells compared with non-cancerous prostate cells. Two independent research groups generated CREB3L4 knock-out mice, and both reported these mice to be healthy and fertile. Their findings using these murine models are slightly different between each other, but they both point to mild defects in spermatogenesis. More specifically, it was found that acetylated H2A and H4 histones are abnormally retained in epididymal sperm, implying that CREB3L4 might be regulating sperm head nuclei maturation in the mouse (Nagamori et al., 2006).

## CREB3 Family in the Central Nervous System

**FIGURE 2:**
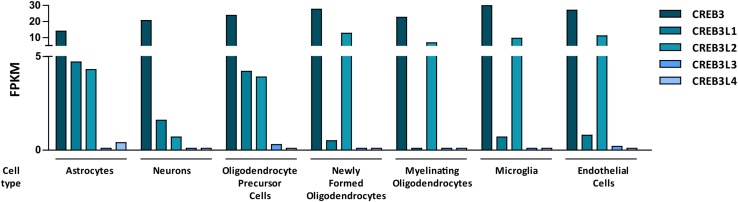
Bar graph showing mouse RNA levels of the five members of the CREB3 family in different cells types from the central nervous system. Data are expressed as FPKM (Fragments per kilo base per million mapped reads). The graph was made with data extracted from a research article by Zhang et al. (2014).

An RNA-sequencing transcriptome study performed with mouse brain cells (Zhang et al., 2014) indicated that CREB3L3 and CREB3L4 are minimally detected in different cell types of the CNS, while CREB3, CREB3L1 and CREB3L2 are co-expressed in most of them (Figure 2). In this section we review their role in physiological and pathological processes of the CNS.

### CREB3

CREB3 is the family member with the highest levels of expression in different cell types of the nervous system (Zhang et al., 2014, Figure 2). As we mentioned above, CREB3 was identified for its interaction with the transcriptional co-activator HCF (Freiman and Herr, 1997; Lu et al., 1997). During herpes virus infection, CREB3 and HCF together with the virion transactivator factor, VP16 induce expression of virion genes. Moreover, it has been postulated that, in neurons of the trigeminal ganglia, CREB3 is required for the establishment of herpes virus latency (Lu and Misra, 2000). Interestingly, CREB3 is also able to activate promoters of genes critical for herpes virus reactivation suggesting a complex role of the CREB3-HCF interaction in this process. The complexity of the CREB3-HCF interaction is harder to understand in dorsal root ganglion neurons where HCF and CREB3 poorly colocalized and exhibited Golgi and ER patterns, respectively (Kolb and Kristie, 2008). Another link between CREB3 and viral infection in the CNS is provided by the CREB3-Herp (homocysteine-induced ER protein) pathway in the Poliovirus (PV)-induced apoptosis. Herp is a CREB3-direct transcriptional target (Liang et al., 2006) involved in Ca2+ regulation in neurons. Down-regulation of CREB3 or Herp expression in IMR5 cells, a neuroblastoma cell line, increased PV-induced apoptosis (Mirabelli et al., 2016). Further analysis will be necessary to understand the participation of CREB3 and Herp in maintaining the balance of pro and antiapoptotic signals during the PV-induced neuropathogenesis of poliomyelitis.

Furthermore, CREB3 regulates intrinsic elongating form of axonal growth linked to injury-associated axonal ER responses (Ying et al., 2014). In dorsal root ganglion sensory neurons CREB3 localizes to the soma and the axonal ER. In response to nerve injury, CREB3 is synthesized in axons and transported to the nuclei of injured neurons via importin-dependent retrograde transport (Hasmatali et al., 2019). Interestingly, immunoprecipitation assays indicated that CREB3 interacts with importin, but CREB3-target genes involved in axon growth were not identified.

CREB3 knock-out mouse model (Penney et al., 2018) used to study the hypothalamic pituitary adrenal (HPA) axis and the glucocorticoid (GC) response linked CREB3 function in CNS to the secretory pathway. These animals have low levels of corticosterone and high levels of the glucocorticoid receptor (GR). Chromatin immunoprecipitation assays performed with mouse embryonic hippocampal cells indicated that CREB3 binds to the promoter region of genes that contain GC response elements (GRE). Interestingly, CREB3 also acts as a GR co-factor since it interacts with GR and enhances GR activity. Furthermore, in hippocampal cells, CREB3 binds to the promoter of genes encoding COPII components, regulating their expression.

### CREB3L1

CREB3L1 expression is transiently up-regulated in the brain of mouse embryos and becomes weaker in the adult (Saito et al., 2012). Up-Regulation of its expression was also demonstrated in reactive astrocytes proximal to a spinal cord injury (Nikaido et al., 2002). Studies performed in CREB3L1 knock-out mice indicated that, in astrocytes, CREB3L1 promotes glial scars formation, impedes axon growth and functional recovery after spinal injury (Sumida et al., 2018). Furthermore, CREB3L1 protein expression was detected in astrocytes and in neuronal primary cultures obtained from hippocampi of mice, but its mRNA up-regulation was detected only in astrocytes after treatment with kainic acid (KA). However, pyramidal neurons in the hippocampi of CREB3L1−/− mice were more susceptible to the toxicity induced by KA than those of wild-type mice (Chihara et al., 2009) suggesting a protective role of astrocytes against the KA-induced neuronal damage. One CREB3L1 target identified in astrocytes is the chondroitin 6-*O*-sulfate transferase 1 (C6ST1) gene, which encodes the major sulfotransferase of proteoglycan chondroitin sulfate (CSPG, Okuda et al., 2014). *In vitro* luciferase assays indicated that CREB3L1 binds to the first intron region of mouse C6ST1 gene. In contrast to the protective role that astrocytes play against KA-induced neuronal damage, astrocytes derived from wild-type CREB3L1 mice inhibited neurite outgrowth of cultured hippocampal neurons, whereas astrocytes from CREB3L1 knock-out mice did not. Therefore, CREB3L1 induction in reactive astrocytes in the injured brain may help to establish a non-permissive microenvironment for regenerating axons (Okuda et al., 2014). Another example of changes in the levels of CREB3L1 that modulate the function of the nervous system is given in the human retinal pigment epithelial cells, ARPE-19 (Miyagi et al., 2013). In these cells CREB3L1 transcriptionally regulates the vascular endothelial growth factor-A (VEGFA) which not only acts as a mediator of angiogenesis but also as a trophic and protective factor of retinal neurons (Foxton et al., 2013).

Increased CREB3L1 expression was also shown in the hypothalamus, specifically in the supraoptic and paraventricular nuclei of dehydrated and salt-loaded rats. The CREB3L1 up-regulation occurs in arginine vasopressin (AVP) neurons, where CREB3L1 activates AVP gene transcription *in vivo* (Greenwood et al., 2014, 2015). In the mouse pituitary cell line AtT20, CREB3L1 expression is up-regulated by cAMP *in vitro*, and orphan nuclear receptor Nr4a1 is the transcription factor controlling the expression of CREB3L1. Furthermore, the ability to activate CREB3L1 by Nr4a1 is related to the level of methylation of the CpG island within the CREB3L1 proximal promoter (Greenwood et al., 2017).

Moreover, a direct link between CREB3L1 levels and changes in neurons was shown in Drosophila class IV dendritic arborization (C4da) neurons where nuclear polyglutamine (polyQ) toxicity reduced CREB3L1/CREBA levels. Additionally, polyQ led to down-regulation of genes involved in the secretory pathway and to the loss of Golgi outposts (GOPs). Furthermore, C4da neurons exhibited defective terminal dendrite elongation and decreased supply of plasma membrane (Chung et al., 2017). In C4da neurons, CREB-binding protein directly regulates CrebA transcription in cooperation with the Drosophila transcription factor Cut (Chung et al., 2017). Consistent with the data obtained in Drosophila, rat hippocampal primary neurons exhibited reduced number of dendrites spines and GOPs due to polyQ toxicity. Also, CREB3L1/CrebA overexpression restored the loss of GOPs and the down-regulation of COPII-related genes induced by polyQ. However, no significant changes exist in the branching and elongation of terminal dendrites for the overexpression of CREB3L1 alone.

### CREBL2

Expression of CREB3L2 *in vivo* was analyzed by immunohistochemistry in a mouse model of permanent focal brain ischemia where CREB3L2 was detected in the region closer to the infarction region in the striatum, especially in neurons (labeled with MAP2). In contrast to CREB3L1, no expression of CREB3L2 was detected in astrocytes in this model. The role of CREB3L2 in the brain was studied using SK-N-SH cells, a human neuroblastoma cell line. Overexpression and siRNA transfection assays indicate that CREB3L2 reduces and increases the sensitivity to ER stress-induced cell death, respectively (Kondo et al., 2007). Although CREB3L2-depleted cells die more than control cells by the action of thapsigargin, the levels of typical ER-stress markers such as GRP78/BiP, XBP1, CHOP, and PDI were not modified. CREB3L2-target genes that protect against cell death induced by thapsigargin were not explored in SK-N-SH cells. Participation of CREB3L2 in cell survival was also reported in mouse malignant glioma GL261 cells (Sheng et al., 2010), where CREB3L2 was identified as one of the 12 genes required for expression of ATF5 (activating transcription factor 5), an anti-apoptotic factor which plays a role in cell survival (Monaco et al., 2007). Sheng et al. (2010) also showed that expression of CREB3L2 in human malignant glioma was higher than in normal brain and that individuals with ATF5-positive glioblastomas had shorter survival times than those with ATF5-negative glioblastomas. Moreover, expression of CREB3L2, ATF5 and its target, the oncogene MCL1 (myeloid cell leukemia sequence 1, Le Gouill et al., 2004) decreased after serum-induced GS9-6 differentiation indicating that these proteins are enriched in undifferentiated cells. ATF5 is required for terminal differentiation and survival of olfactory sensory neurons according to homozygous Atf5 knock-out mice (Wang et al., 2012).

CREB3L2 levels also increase during oligodendrocytes (OL) maturation where Chd7 (chromodomain-helicase-DNA-binding protein 7) and Sox10 activates a transcriptional program for OL differentiation (He et al., 2016). CREB3L2 and Osterix are Chd7 targets and their down-regulation in OL precursor cells inhibited expression of genes related to myelination (He et al., 2016). Moreover, in the dorsal-root ganglion neurons-like F11 cell line, CREB3L2 was identified as a direct target gene of the transcription factors nuclear factor IL-3 (NFIL3) and the tree isoforms of the CCAAT-enhancer-binding proteins (C/EBPα, C/EBPβ, and C/EBPδ; Macgillavry et al., 2011). It has been postulated that these transcription factors co-regulate CREB3L2 expression during forskolin-induced neurite outgrowth model. NFIL3 and C/EBPs integrate a complex transcriptional regulatory network that fine-tunes the expression of neuronal outgrowth-related genes.

## Outlook

**FIGURE 3:**
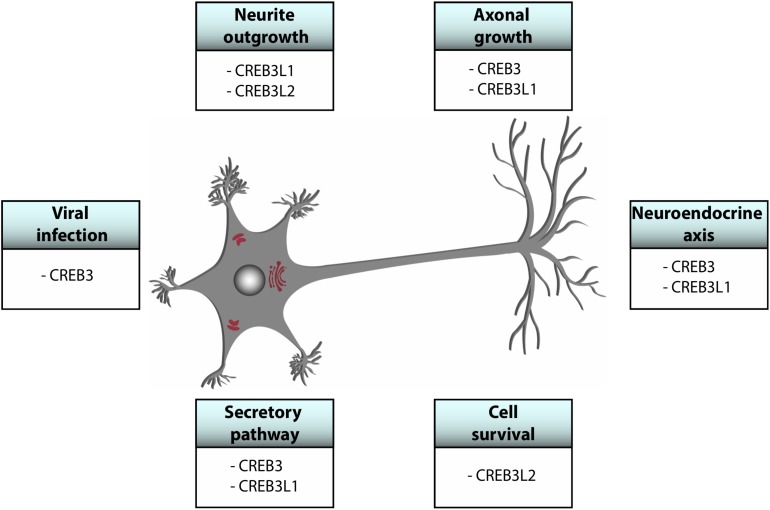
Schematic representation of global functions of CREB3 proteins in the central nervous system.

CREB3 transcription factors are widely expressed in different tissues and they regulate a broad range of developmental, physiological and pathological processes playing fundamental roles in cellular homeostasis. Most of them show a tissue-specific preferential expression, however, CREB3, CREB3L1, and CREB3L2 co-express in different cells of the CNS (Figure 2) where they participate in essential processes (Table 1 and Figure 3). For example, CREB3L1 and CREB3L2 are involved in neurite outgrowth (Macgillavry et al., 2011; Okuda et al., 2014) while CREB3 and CREB3L1 modulate (directly or indirectly) axonal growth after an injury (Ying et al., 2014; Sumida et al., 2018). CREB3 regulates pathogenic mechanisms of herpes and polio virus (Lu and Misra, 2000; Liang et al., 2006), and in non-CNS cells a CREB3-ARF4 signaling pathway mediates the susceptibility to pathogens (Reiling et al., 2013). As we mentioned above, CREB3 transcription factors can also modulate cell survival by activating expression of anti-apoptotic factors (Sheng et al., 2010). Also, CREB3 and CREB3L1 contribute to neuroendocrine regulation of the hypothalamic/pituitary/adrenal axis modulating the GR activity and the AVP gene transcription (Greenwood et al., 2014; Penney et al., 2018). Most of these processes require the adaptation of the secretory pathway which is regulated by CREB3 family members in multiple cell types (Fox and Andrew, 2015). In line with this, CREB3 regulates expression of genes encoding COPII components and formation of Golgi outposts in hippocampal cells (Chung et al., 2017; Penney et al., 2018).

**TABLE 1:** CREB3 family members in the central nervous system, list of their functions, upstream regulators and downstream target genes.

**Family member**	**Cell type/Tissue/Animal model**	**Function/s**	**Upstream regulator**	**Target gene**	**References**
	Neuroblastoma cell line (IMR5)	Balance of apoptotic and anti-apoptotic signals during the Poliovirus-induced poliomyelitis	N/D	Homocysteine-induced ER protein (Herp)	Liang et al., 2006; Mirabelli et al.,2016
	Dorsal root ganglion neurons (mouse)	Regulates axonal growth	N/D	N/D	Ying et al.,2014
CREB3	Neurons in the trigeminal ganglia	Establishment of herpes virus latency	N/D	IE110 gen, required for virus reactivation	Lu and Misra,2000
	CREB3 knock-out mouse model	Modulates glucocorticoid response	N/D	Genes containing glucocorticoid response elements (GRE)	Penney et al.,2018
	Primary cultured neuronal precursor cells from mice Cerebral cortices of mice	Differentiation of astrocytes form NPCs Demethylation of the Gfap promoter	N/D	Gcm1 (direct)	Saito et al.,2012
CREB3L1	Human retinal pigment epithelial cells (ARPE-19)	Transcriptional regulation of VEGF. Indirect modulator of retinal neurons function.	N/D	Vascular endothelial growth factor-A (VEGFA)	Miyagi et al.,2013
	Arginine vasopressin (AVP) neurons		Orphan nuclear receptor Nr4a1	Arginine vasopressin (AVP)	Greenwood et al.,2017
	CREB3L1 knock-out mouse model	Establish a non-permissive microenvironment to regenerating axons	N/D	Chondroitin 6-O-sulfate transferase 1 (C6ST1)	Okuda et al.,2014
	Drosophila class IV dendritic arborization (C4da) neurons	Golgi Outpost Synthesis	CREB-binding protein (CBP) and Cut	COPII pathway related genes	Chung et al.,2017
	Human neuroblastoma cell line (SK-N-SH)	Protects against cell death induced by thapsigargin	N/D	N/D	Kondo et al.,2007
	Mouse malignant glioma cells (GL261)	Activate expression of ATF5-regulated antiapoptotic genes (myeloid cell leukemia sequence 1, MCL1)	N/D	ATF5 (activating transcription factor 5)	Sheng et al.,2010
CREB3L2	Dorsal-root ganglion (DRG) neurons-like cells (F11)	Expression of neuronal outgrowth-related genes	Nuclear factor IL-3 (NFIL3) and CCAAT-enhancer-binding proteins (C/EBPs)	N/D	Macgillavry et al.,2011
	Primary rat Oligodendrocytes and/or their precursors	Contribute to Oligodendrocyte differentiation	Chromodomain-helicase-DNA-binding protein 7 (Chd7)	N/D	He et al.,2016

*N/D, not defined.*

CREB3 transcription factors emerge as signaling hubs for the regulation of Golgi homeostasis, integrating stimuli from multiple sources to control secretion, protein post-translational modification and trafficking, impacting on membrane expansion and composition. Their contribution to the CNS is crucial since neurons are specially sensitive to Golgi stress and Golgi fragmentation, events tightly connected to neurodegenerative diseases (Gonatas et al., 2006; Machamer, 2015; Lopez et al., 2017). Although some upstream regulators and downstream target genes of CREB3 transcription factors have been identified (Table 1), many questions remain open: What are the specific stimuli that trigger the activation of these factors and how are they sensed? How are CREB3 family genes regulated in each cell type of the CNS? What are the consequences of lacking one or more CREB3 transcription factors in a cell? Can they replace each other? How do they participate in neuronal development? Comprehensive understanding of how CREB3 transcription factors function promises not only to explain fundamental biological questions, but also to provide new options for therapeutic intervention.

## Author Contributions

CA, LS, and PD wrote the manuscript. CA contributed with funding support.

## Conflict of Interest Statement

The authors declare that the research was conducted in the absence of any commercial or financial relationships that could be construed as a potential conflict of interest.
